# Use of research electronic data capture (REDCap) in a sequential multiple assignment randomized trial (SMART): A practical example of automating double randomization

**DOI:** 10.21203/rs.3.rs-2573133/v1

**Published:** 2023-02-24

**Authors:** Carol A. Lee, Danilo Gamino, Michelle Lore, Curt Donelson, Liliane C. Windsor

**Affiliations:** Addiction Center, University of Michigan, North Campus Research Complex Building 16, 2800 Plymouth Rd., Ann Arbor, MI 48109, USA; North Jersey Community Research Initiative, 393 Central Ave., Newark, NJ 07103; Interdisciplinary Health Sciences Institute, University of Illinois at Urbana Champaign, 901 West University Ave., Urbana, IL 61801; Data and Technology Innovation Group, University of Illinois at Urbana Champaign, 901 West University Ave., Urbana, IL 61801; School of Social Work, University of Illinois at Urbana Champaign, 1010 W. Nevada St., Urbana, IL 61801

**Keywords:** Research Electronic Data Capture (REDCap), randomized controlled trials (RCT), adaptive interventions, Sequential Multiple Assignment Randomized Trial (SMART), randomization, experimental design, reducing human errors, automation

## Abstract

**Background:**

Adaptive interventions are often used in individualized health care to meet the unique needs of clients. Recently, more researchers have adopted the Sequential Multiple Assignment Randomized Trial (SMART), a type of research design, to build optimal adaptive interventions. SMART requires research participants to be randomized multiple times over time, depending upon their response to earlier interventions. Despite the increasing popularity of SMART designs, conducting a successful SMART study poses unique technological and logistical challenges (e.g., effectively concealing and masking allocation sequence to investigators, involved health care providers, and subjects) in addition to other challenges common to all study designs (e.g., study invitations, eligibility screening, consenting procedures, and data confidentiality protocols). Research Electronic Data Capture (REDCap) is a secure, browser-based web application widely used by researchers for data collection. REDCap offers unique features that support researchers’ ability to conduct rigorous SMARTs. This manuscript provides an effective strategy for performing automatic double randomization for SMARTs using REDCap.

**Methods:**

Between January and March 2022, we conducted a SMART using a sample of adult (age 18 and older) New Jersey residents to optimize an adaptive intervention to increase COVID-19 testing uptake. In the current report, we discuss how we used REDCap for our SMART, which required double randomization. Further, we share our REDCap project XML file for future investigators to use when designing and conducting SMARTs.

**Results:**

We report on the randomization feature that REDCap offers and describe how the study team automated an additional randomization that was required for our SMART. An application programming interface was used to automate the double randomizations in conjunction with the randomization feature provided by REDCap.

**Conclusions:**

REDCap offers powerful tools to facilitate the implementation of longitudinal data collection and SMARTs. Investigators can make use of this electronic data capturing system to reduce errors and bias in the implementation of their SMARTs by automating double randomization.

**Trial registration:**

The SMART study was prospectively registered at Clinicaltrials.gov; registration number: NCT04757298, date of registration: 17/02/2021.

## Background

Research Electronic Data Capture (REDCap) is a secure, browser-based web application available to approximately 2.3 million users in 151 countries.^1^ The application is primarily used for developing, maintaining, and managing different types of surveys and securing online/offline data collection. REDCap also offers useful features like a randomization module that can be valuable for conducting randomized controlled trials (RCTs). The randomization module in REDCap helps researchers implement a defined randomization model within their project, allowing them to randomize participants (i.e., records in their project). The module also monitors the overall allocation progress and assignment of randomized participants.

Biological and behavioral interventions delivered by clinicians in the real world typically require a sequential, individualized approach in which interventions like prevention and treatment strategies are adapted and re-adapted over time in response to the specific needs and evolving status of the client. For instance, suppose a clinician starts a client who is suffering from depression in psychotherapy, an evidence-based intervention. After 30 days, the client has not experienced any improvement and has not been attending sessions consistently. The clinician needs to decide whether to continue the psychotherapy or add medication, another evidence-based intervention, to the treatment plan.

Most health research interventions have focused on testing individual interventions like psychotherapy or specific medications in a specific dose and format which are then offered to all program participants.^2–6^ Under this approach, an intervention must be delivered to all clients in the same format and dosage, regardless of individual reactions.^7^ Notably, this approach does not offer evidence-based guidance about when to change an intervention, which strategy works best for different subpopulations, or how to dose the combinations of possible interventions.

Recently, adaptive interventions have emerged as a new means of providing research-based prevention and treatment.^8,9^ Adaptive interventions recognize that the varying intervention needs of individuals may not be optimally met via a uniform composition and dosage. For this reason, an adaptive intervention assigns different dosages of certain program components to different individuals, and/or different dosages to the same individuals over time. Dosage varies in response to the intervention needs of clients, and dosages are assigned based on decision rules linking characteristics of the individual client with specific levels and types of program components. In some adaptive interventions, a dosage of zero is possible for a given component, meaning that some individuals might not receive certain components at all, and that different types or versions of program components may be assigned to different individuals.^8,9^ Part of the conceptual appeal of the adaptive approach is its clear resemblance to clinical practice, which tailors treatment to individuals based on their unique needs, which can change over time.

Sequential Multiple Assignment Randomization Trial (SMART) was created as a research strategy to develop and test adaptive interventions. It was first introduced as “biased coin adaptive within-subject designs” by Lavori and Dawson, with the general framework proposed by Murphy.^10^ Given the increasing interest in personalized medicine among general population and field of public health, over the last decade SMARTs have become a more common and streamlined fixture of the clinical trial landscape.^11^ The SMART approach is a randomized experimental design developed especially for building time-varying adaptive interventions.^12,13^ Developing such an adaptive intervention strategy requires addressing questions such as:

- What is the best sequencing of intervention components?
- Which tailoring variables should be used?
- How frequently, and at what times, should tailoring variables be reassessed and an opportunity for changing amount and/or type of intervention be presented?
- Is it better to assign treatments to individuals, or to allow them to choose from a menu of treatment options?

The SMART approach enables intervention scientists to address such questions holistically and rigorously by considering the order in which intervention components are presented rather than considering each intervention component in isolation. In this way, a SMART approach provides an empirical basis for selecting appropriate decision rules and tailoring variables, with the end goal of developing evidence-based adaptive intervention strategies to be evaluated in subsequent RCTs.^13^

**Figure 1.**
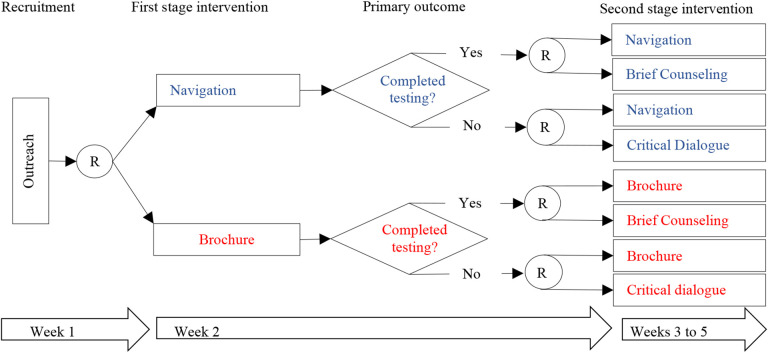
COVID-19 Optimization Study Design Using SMART Method. *Navigation: NS include assessment and support with service referrals. Each participant will meet with a peer navigator in person or on Zoom conferencing for 30 min to go over the results from their social and health needs assessment that is retrieved from the baseline survey. The navigator shares information about COVID-19, answers questions about testing, and makes referrals to other needed services. Follow-up sessions occur as needed. *Brochure: A digital brochure will consist of a brochure containing CDC public health recommendations that is e-mailed or tested to participants immediately after randomization. The brochure includes information about COVID-19, testing, and vaccines. *Critical dialogue: CD includes three 1 hour long, open-group sessions facilitated in person or online by a trained licensed facilitator. Group critical dialog is promoted by thematic images to foster a deeper understanding of how systematic stigma, feelings of rage as victims of discrimination, and/or apathy may impact participants’ beliefs and behaviors related to COVID-19 and empower participants to make critical choices to protect their health and the health of their communities. *Brief counseling: BC is a 15 minute post-COVID-19 test session delivered by a trained licensed clinician in person or via Zoom conferencing. In the session, the clinician shares the test results and offers recommendations and information about COVID-19 treatment and prevention.

There are two common SMART designs: (a) all participants are re-randomized following initial treatment; and (b) only participants who failed to respond to their initial treatment are re-randomized and the rest continue their initially assigned treatment. In Figure 1, a circled R indicates randomization, and a box indicates a particular treatment. Although more complex methods are required to estimate more deeply tailored regimen, simple regimen that tailor only in the variable used to determine second-stage randomizations can be easily conducted using intention-to-treat analysis, in which the intended treatment is determined by the outcome of the randomization.

In both designs, SMARTs are characterized by various treatment sequences. Automated randomizations can save time and reduce human error that can impede researchers’ ability to follow participants and engage them in research. However, automated implementation software solutions for SMARTs remain scarce. One significant challenge for SMART design study is the availability of affordable and accessible software capable of conducting automated randomization.^14^ The current paper uses a case study^15^ to address this gap by describing the development of a strategy that successfully automatically randomized participants multiple times to different interventions using Research Electronic Data Capture (REDCap) in conjunction with an application programming interface (API), based on a priori criteria regarding participants’ responses to interventions over time. We also provide statistical codes that future researchers can easily adapt to meet the unique needs of their individual projects.

## Methods

### Study Case Description

The rapid worldwide spread and impact of COVID-19 underlined the need for interventions that effectively increase adherence to public health recommendations (e.g., testing, vaccination, social distancing, and mask wearing). Returning safely to pre-pandemic routines and practices depends on governments’ and providers’ ability to streamline the delivery of these effective interventions to the individuals who need them. To this end, the National Institutes of Health (NIH) launched the Rapid Acceleration of Diagnostic-Underserved Populations (RADx-UP) program, designed to ensure that all Americans have access to COVID-19 testing, with a focus on communities disproportionately affected by the pandemic. Projects funded by RADx-UP include new applications of existing technologies that make tests easier to use, easier to access, and more accurate. As delineated in the study protocol document^15^, this study case was a part of the RADx-UP network of projects.

This study case used a SMART with 670 people who are medically/socially vulnerable to COVID-19 to: (a) optimize an adaptive intervention to increase rates of COVID-19 testing among high-risk populations; and (b) identify predictors of COVID-19 testing uptake. For the primary aim of this parent study, standard sample size formula for the difference of two proportions, a total sample size of 582 subjects was needed to have 80% power at a = 0.05 to detect a 10% difference in proportions, assuming 70% in the brochure group will complete testing. The 582-sample size had 85% to detect a difference of 15% in testing rates between the two interventions. The study was not powered to detect changes in the second-stage interventions. Figure 1 shows the SMART design implemented of the study. The study took place at the North Jersey Community Research Initiative (NJCRI, www.njcri.org) located in Newark, Essex County, NJ. Clinical emergencies were handled by the NJCRI treatment team of experienced staff according to the study protocol. Appropriate assessments were conducted; treatment options were recommended and followed through as necessary.

The following were the inclusion criteria:

- Over 18 years of age
- Having a high risk to contract COVID or develop related complications
- Able to speak English
- Able and willing to provide informed consent

The following were the exclusion criteria:

- Under 18 years of age
- Not at a high risk to contract COVID or develop related complications
- Unable to speak English
- Unable or unwilling to provide informed consent

A hallmark of the SMART design is that it requires multiple randomizations at multiple timepoints. For instance, consider the current COVID-19 Optimization Trial study case, which seeks to increase COVID-19 testing in a marginalized community. Research questions in this trial include:

- Will navigation services (NS) result in higher rates of COVID-19 testing compared to an electronic informational brochure?
- For those who complete testing, will continuing NS intervention result in higher rates of adherence to CDC recommendations than switching to brief counseling?
- For those refusing to get tested, will continuing with information brochure intervention result in more people subsequently getting tested, compared to switching to critical dialogue intervention?

Clients are randomized for the first time to *Navigation* or *Brochure* treatments and then, at one week, randomized again to continue the original intervention or switch to a different approach. By randomizing each of the initial treatment groups, researchers can obtain a cohort of participants who followed each of the four possible treatment regimens (i.e., *Navigation Only*, *Navigation with Brief Counseling*, *Brochure Only*, *Brochure with Brief Counseling*), allowing a direct comparison of the effects of each regimen. The four possible treatment regimens are termed *embedded regimen*.

### Randomization Challenge

The REDCap software includes a platform for randomization. This module allows researchers to perform simple or stratified randomization, and to randomize participants by site in multi-site studies. Once the randomization model is established, the software also provides allocation table templates as an example for researchers to create their own allocation tables. Following a project’s move to *production* mode, REDCap locks the randomization model, ensuring it is not modified after a study becomes active. However, although the existing REDCap randomization model is robust, it cannot accommodate two randomization models within one project, making it difficult to perform a double randomization within one REDCap project. Concerningly, we could identify no other affordable software solutions capable of including multiple randomization models.

By consulting with REDCap administrators from other institutions on the REDCap Community Site – a message board where REDCap administrators from around the world can confer with one another to troubleshoot and develop innovative solutions – our research team identified one workaround to this limitation: namely, to create two separate REDCap projects, one for the first randomization and one for the second randomization. However, this model would require migrating data between projects, which increases the likelihood of accidental data loss or invalidation. The Illinois REDCap administrators provided two alternative suggestions. The first was to perform one randomization using the REDCap randomization module and the other randomization external to REDCap and manually enter the randomization result. The second solution was to write application programming interface (API) code for conducting one or both randomizations outside of REDCap. The research team decided to incorporate the REDCap API into the final suggestion, thus conducting both randomizations outside of REDCap and automating the entry of the randomization results generated outside of REDCap.

### Implementing the Double Randomization

The technical implementation of the double randomization algorithm involved three services: the REDCap API, Amazon Web Services (AWS) Lambda, and AWS S3. The REDCap API is a programmatic interface that allows for the controlled movement of data between REDCap and other software; Lambda functions are short scripts that can run quickly and automatically within AWS when certain trigger conditions are met; and S3 is a file-storage system within AWS that can work in tandem with other AWS services (e.g., Lambda) to efficiently provide and receive files in any format. To accomplish the double randomization, two processes (described in the next paragraphs) automatically ran every morning. The processes operated similarly but had different inclusion criteria and randomization factors.

The first randomization began with a time-triggered Lambda function that called the REDCap project via the API and queried for a list of subjects that had consented, been approved by research staff, and had not yet received their first randomization. The REDCap API then returned truncated records to the Lambda function containing record IDs and the necessary inclusion criteria. The same Lambda function then queried a specific S3 bucket and fetched an allocation table in csv format. This table was created by the project’s statistician and contained a randomized list of first outcomes. The list of subjects was then stepped through, and each subject was assigned the outcome at the top of the table. After an outcome was assigned, it was moved to the bottom of the table so that it would not be assigned again until the entire table had been exhausted. Once every subject from that day had been assigned an outcome, the updated allocation table was pushed back to its location in S3 for use on the next day and the subject data were imported back into REDCap via the API.

The second randomization followed a similar process as the first but had more inclusion criteria. After the first randomization Lambda function was complete, a second Lambda function then queried the same REDCap project and fetched subjects who had been assigned a first randomization outcome and had reached their deadline for completing a COVID-19 test but had not yet received a second randomization outcome. The Lambda function then queried the same S3 bucket but fetched a different allocation table than the first. The second allocation table contained outcomes for the second randomization but was slightly more complex because there were four possible outcomes with different inclusion criteria based on their first randomization status. For each eligible participant, the table was read sequentially until a row was found that matched the participant’s criteria, including first randomization status, COVID-19 test status, and test result. This row’s outcome was attached to the subject’s data, and then moved to the bottom of the table in the same way as in the first allocation process. After all eligible participants had been assigned a second randomization, the allocation table was pushed back to the S3 bucket, and the subject data was imported back into REDCap via the API.

Our research team decided to write an API code for both randomizations to be conducted outside of REDCap and to automatically indicate the result of randomization for each participant in REDCap. The first randomization runs automatically at 3:00 a.m. It pulls in all records where:

*approve_b* variable equals “Yes”
AND
*consent_2* variable equals “Yes”
AND
*randomization1* variable is blank

The second randomization runs automatically at 3:05 AM. It pulls in all records where:

*randomization1* variable is not blank
AND
*randomization2* variable is blank
AND
*covid_test* variable is not blank
AND
*covid_test_complete* variable equals “Yes”

The second randomization pulls anyone who has received the first randomization but not the second, has reported “Yes” or “No” on taking a COVID-19 test, and has a COVID-19 test instrument marked “complete.” The code checked every morning for records requiring randomization, and if it found none then no further action would be performed that day.

The main challenge of this type of programmatic solution was keeping the code in sync with the up-to-date REDCap project format. Changes in the project instruments, or certain variables within the instruments, required manually updating the code inside the Lambda functions to ensure that the data import would complete. Another concern was that the growing size of the subject population meant a longer Lambda function runtime. Although runtime never approached the maximum timeout limit in the present study, we had to make occasional adjustments to ensure that the processes successfully ran every morning. Limitations on Lambda scaling means that studies with exceptionally large populations may cause the code to run past the allotted time limit.

## Results of Double Randomizations

**Figure 2.**
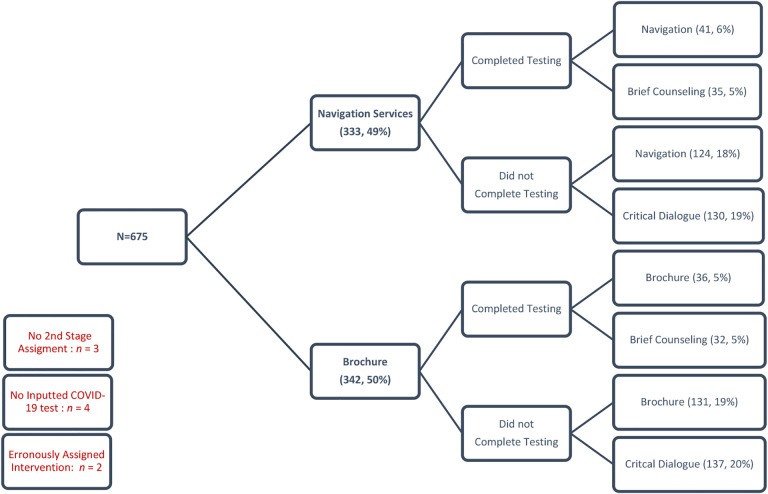
Summary of sequential multiple randomizations results.

Overall, despite a few errors, the randomization strategy was very successful, saving the staff substantial time and minimizing randomization and data entry errors. In total, 675 participants met all the inclusion criteria and none of the exclusion criteria. All 675 participants have been successfully randomized to their first randomization assignment. Figure 2 shows the randomization results. A total of 333 (49.3%) were assigned to “Navigation Services” and 342 (50.7%) to “Brochure.” Participants needed to report whether they have completed a COVID-19 test in order to be included in a second round of randomization. Their covid-19 status is assessed by study staff 7 days after being enrolled either by phone or in-person, and responses are manually entered into the REDCap database. Only 22.2% (*n* = 151) of participants took the COVID-19 test, and 77% (*n* = 528) reported not taking the COVID-19 test.

Participants who were initially randomized to “Navigation Services” underwent second randomization as follows: among those who completed COVID-19 testing, 6% (*n* = 41) were randomized to “Navigation Services” and 5% (*n* = 35) were randomized to “Brief Counseling.” Among those who did not complete COVID-19 testing, 18% (*n* = 124) were randomized to “Navigation Services” and 19% (*n* = 13) were randomized to “Critical Dialogue.”

Participants who were initially randomized to “Brochure” underwent second randomization as follows: among those who completed COVID-19 testing, 5% (*n* = 36) were randomized to “Brochure” and 5% (*n* = 32) were randomized to “Brief Counseling.” Among those who did not complete COVID-19 testing, 19% (*n* = 131) were randomized to “Brochure” and 20% (*n* = 137) were randomized to “Critical Dialogue.”

The second randomization failed for nine participants (equivalent to 1.3% error): three participant IDs were missing second randomization assignments after these participants dropped out before the second round of randomization occurred; four participant IDs had no COVID-19 test information inputted, and therefore they were never assigned a second intervention; and two IDs were accidentally manually assigned to a different intervention by a study staff member.

Because the randomization process occurred daily, in some instances the automatically assigned interventions were delivered two to three days after they were due to be delivered. These delays occurred when the randomization assignments were generated on weekends when study staff were not available to check the system and deliver the intervention within the required 24-hour window post-randomization. To mitigate this issue, we limited the REDCap entry of the two key variables (i.e., *approve_b* and *consent_2*) needed to start the randomization process limited from Mondays through Thursdays. This gave the study staff time within the work week to deliver the assigned interventions, avoiding potential delays.

## Discussion

The current paper used a case study to demonstrate how SMART design researchers can conduct multiple randomizations despite the lack of accessible software capable of conducting automated multiple randomizations. Namely, we illustrated a strategy that our research team successfully used to automatically randomize participants multiple times to different interventions using REDCap.

**Figure 3.**
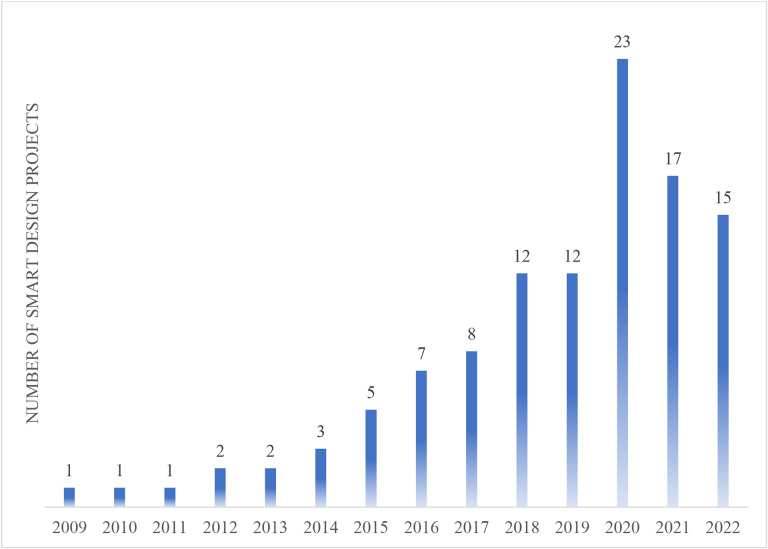
NIH-funded SMART design projects by year.

As adaptive interventions have emerged as a new approach to research-based prevention and treatment, SMART designs have been gaining popularity. According to the NIH’s Research Portfolio Online Reporting Tools (RePORT) website, which allows users to search a repository of both intramural and extramural NIH-funded research projects and access publications and patents resulting from NIH funding, 109 NIH-funded projects used SMART design during the 2022 calendar year. The Figure 3 shows a steady increase in NIH-funded projects employing a SMART design since 2009.

As SMART design gains increasing recognition and is increasingly implemented in practice, demand for software capable of performing the unique multiple randomization assignments required for SMARTs will also increase. Encouragingly, our strategy of utilizing API for the double randomization procedure was seamlessly implemented in conjunction with the REDCap platform. This strategy eliminated technological and logistical challenges by effectively concealing and masking intervention allocation sequences to investigators, involved health care providers, and subjects as they progressed through the complex SMART study procedures. At the same time, challenges common to all rigorous clinical trials (e.g., study invitations, eligibility screening, consenting procedure, data collection, and data confidentiality protocols) were addressed adequately through unique features of the REDCap platform. Additional benefits of using API in conjunction with REDCap for our SMART design project included: timely completion of complex intervention assignments, accurate assignments for participants at multiple time points, and the ability to reduce both the human resources required to implement the study and the likelihood of human error by automating most of the study procedures.

Although this strategy worked very well for our research team, some contextual factors might influence how other researchers adapt the strategy to their projects in different organizations. The global REDCap community may develop an add-on that accomplishes multiple randomizations within a single project. However, there may be a lag between its release and the ability of organizations to deploy it on different REDCap instances. For example, the University of Illinois at Urbana-Champaign’s REDCap instance, which housed our project, had a built-in Health Insurance Portability and Accountability Act (HIPAA)-compliant AWS account. The University also takes steps to guarantee the security of the software. Most importantly, the Illinois REDCap instance undergoes an annual security review with the university’s Cybersecurity Governance, Risk, and Compliance (GRC) team. The review evaluates Illinois REDCap’s ability to meet a variety of security controls and measures in accordance with HIPAA and the University’s cybersecurity standards. In addition to the annual review, REDCap users are required to complete HIPAA training before accessing the system, system sign-on utilizes Shibboleth two-factor authentication, and projects are vetted by REDCap administration team for required protections before a project is moved to production to ensure security. Thus, due to the secure nature of Illinois REDCap, our process of integrating third-party solutions within REDCap was rigorous. The add-on was inspected for compliancy and potential vulnerabilities, then tested on a TEST server. Additionally, documentation and standard operating procedures for how the add-on will be upgraded and accessed by users were developed by the University.

The unique context of each organization must be taken in consideration before implementing this multiple randomization solution. Whereas our research team was able to successfully utilize the built-in HIPAA-compliant AWS account to implement our strategy in conjunction with rigorous REDCap team administration support for data security and protection, other research teams may not have the same resources and administrative support. Some of the ways in which research teams with limited resources to readily implement this strategy may consider consulting REDCap community that is very active in providing recommendations and support.

## Conclusions

REDCap is a free, widely available data collection system that offers powerful tools for longitudinal data collection. Utilizing an online data collection system for SMART design studies can decrease biases within studies while requiring fewer human resources and decreasing opportunity for human error. Investigators can make use of this electronic data capturing system in conjunction with AIP to successfully complete their SMART design studies.

## Data Availability

The datasets used and analyzed during the current study available from the corresponding author on reasonable request and with permission of University of Illinois at Urbana-Champaign.
